# Design of Refining Slag Based on Structural Modifications Associated with the Boron Removal for SoG-Si

**DOI:** 10.3390/ma15093107

**Published:** 2022-04-25

**Authors:** Guoyu Qian, Yiwei Sun, Dong Wang, Zhiliang Wu, Zhi Wang, Wenhui Ma

**Affiliations:** 1 Key Laboratory of Green Process and Engineering, National Engineering Laboratory for Hydrometallurgical Cleaner Production Technology, Institute of Process Engineering, Chinese Academy of Sciences, Beijing 100190, China; sunyiwei19@mails.ucas.ac.cn (Y.S.); dongwang@ipe.ac.cn (D.W.); 2 Faculty of Metallurgical and Energy Engineering, Kunming University of Science and Technology, Kunming 650093, China; xy42002@163.com (Z.W.); mwhsilicon@126.com (W.M.)

**Keywords:** solar grade Si, slagging treatment, B removal, raman spectroscopy, NMR spectroscopy

## Abstract

Solar grade silicon (SoG-Si) is the core material of solar cells. The removal of boron (B) has always been a challenge in the preparation of high purity Si. Slag refining has always been considered as one of the effective methods to remove B, but the design of refined slag has been limited by the cognition of the relationship between slag structure and impurity removal, and can only rely on the apparent basicity and oxygen potential adjustment of slag based on a large number of conditional experiments. In order to clarify the *B* removal mechanism of slag refining from Si, nuclear magnetic resonance (NMR) and Raman vibrational spectroscopy were used to investigate in detail the behavior and state of B and aluminum (Al) in the SiO_2_–CaO–Al_2_O_3_–B_2_O_3_ slag. The role of the degree of B–Si cross linking on the B activity in slag was highlighted by comparing the partition ratio (*L*_B_) between slag and Si. *Q*^2^ structural unit of slag is an important site for capturing B. BO_4_ (1B, 3Si) species is the main form of connection between B and silicate networks, which determines the activity of B in the slag. The addition of Al_2_O_3_ into SiO_2_–CaO slag can change the relative fraction of *Q*^2^ and BO_4_ (1B, 3Si). Increasing Al_2_O_3_ content from 0 to about 20 wt% can lead to the overall increase of *Q*^2^ population, and a tendency to decrease first and then increase of BO_4_ (1B, 3Si) fraction under both basicity conditions (0.6 and 1.1). When Al_2_O_3_ content is less than 10 ± 1 wt%, the decrease of BO_4_ (1B, 3Si) population plays a major role in deteriorating the connectivity between B and aluminosilicate network, which leads to a higher activity of B. When the Al_2_O_3_ content is greater than 10 ± 1 wt%, B is incorporated into the silicate network more easily due to the formation of more *Q*^2^ and BO_4_ (1B, 3Si), which contributes to a rapid decline in activity of B in slag.

## 1. Introduction

Solar energy is an important clean green energy for our current society, and it can be converted into electricity through photovoltaic modules (PV module) [1,2], where the most important Si raw material is solar grade Si (SoG-Si, 6N-7N) with extremely low content of impurities. B is considered to be one of the most detrimental elements because of its doping effect on Si [3], and its removal to a required range has been proved a very challenging task. Modified Siemens process, a typical chemical method representation, can only produce high purity Si with purity greater than 9N [4]. As a result, B and P have to be doped for the requirement of the solar cell, which would lead to a double waste of resources and energy. The core of chemical purification methods such as Siemens method [5] is to make a series of phase transformation of Si to obtain SoG-Si, resulting in large energy input, a long process, and high cost. The essence of metallurgical method is physical separation; namely, impurities are removed step by step from Si based on its separation properties, which has advantages in terms of energy consumption reduction, cost reduction, and process shortening [6,7,8,9,10]. For example, according to the oxidizing property of B, slag oxidation refining is carried out for the removal of B, vacuum refining is carried out for the removal of phosphorus according to the volatility of phosphorus, and directional solidification is carried out for the removal of metal impurities according to the characteristics of small separation coefficient of metal impurities in Si (the separation coefficient of most metal impurities is less than 10^−3^) [6].

Compared with vacuum refining and directional solidification refining, slag oxidation refining has always been regarded as an effective method to remove B impurities from Si [11,12,13,14]. In the slag refining process, B is oxidized into slag at the interface between slag and Si by reactions (1) [6], so as to realize the removal of B from Si.

(1) $$\left[B\right]+\left(3/4\right)O_{2}=\left(1/2\right)B_{2}O_{3}$$

(2) $$\left(1/2\right)B_{2}O_{3}+\left(3/2\right)O^{2-}={\mathrm{BO}}_{3}^{3-}$$

(3) $$L_{B}=\frac{\left(B\right)}{\left[B\right]}=K\frac{\gamma_{B}a_{O^{2-}}^{3/2}P_{O_{2}}^{3/4}}{\gamma_{{\mathrm{BO}}_{3}^{3-}}}=K\frac{\gamma_{B}a_{O^{2-}}^{3/2}}{\gamma_{{\mathrm{BO}}_{3}^{3-}}}{\left(\frac{K^{0}a_{{\mathrm{SiO}}_{2}}}{a_{\mathrm{Si}}}\right)}^{3/4}$$

where, $\gamma_{B}$ and $\gamma_{{\mathrm{BO}}_{3}^{3-}}$ are the activity coefficients of B in silicon and slag, respectively, $a_{\mathrm{Si}}$ and $a_{{\mathrm{SiO}}_{2}}$ are the activity of Si and SiO_2_, *K* is the is the equilibrium coefficient, $a_{O^{2-}}$ is the oxygen potential of slag, and $L_{B}$ is the partition coefficient of B between slag and Si.

The design of slag composition is the core of slag refining to remove B, which determines the ultimate removal capacity of B impurities. On the basis of determining the limit of impurity removal capacity of slag, methods such as applying physical field or adding alloy medium to Si to change the activity of impurities in Si have been carried out to approach the limit of impurities removal capacity of slag as much as possible [15,16,17,18]. Therefore, the improvement of impurity distribution coefficient through slag composition optimization has become the focus of many scholars. The basicity (being a function of basic oxides activity) [19,20] and oxygen potential ($P_{O_{2}}$ being a function of SiO_2_ activity) [20] have always been key parameters for the design of slag composition. The activity of B in slag is one of the most important thermodynamic parameters, which directly affects the oxidation removal of B from Si. However, the influence of basicity and oxygen potential on the activity of B is often derived from empirical parameters based on a large number of conditional experiments. There is a lack of research on the structure-activity relationship between slag structure and refining effect, which leads to the lack of deep theoretical guidance in a large number of slag-refining research and process demonstration, and cannot get rid of blind exploration and exhaustive research ideas.

Any progress in the development of various subject areas requires first a detailed description of the bulk structure of research object, such as biotechnology [21], foundation chemistry [22], battery materials [23], and so on. The knowledge of these structural factors is a key to greatly improve the development of the refinement of slag. For slag structure, the basis of the coordination environment is the silicate network, and it can be characterized by the bridging oxygens (BO) and non-bridging oxygen (NBO) in each SiO_4_ polyhedron [24,25,26]. Basic oxide can destroy the silicate network to produce *Q^n^* units (*Q^n^* refers to Si with n BOs and 4 − *n* NBOs) [27,28]. Borate as oxygen acceptors can form chemical bonds with other oxides and incorporate into the Si network. A BO connects two adjacent polyhedra, such as Si–O–Si and Si–O–B, determining the connectivity of silicate and phosphate network. For a NBO, it can connect a polyhedra and cation by the form of Si–O–Ca and B–O–Ca, denoting a fragmented network. Moreover, B has an electron configuration of 1s^2^2s^2^2p^1^ outside its nucleus, and it can be hybridized to be sp^2^ or sp^3^ orbitals, which presents a triangle (BO_3_) or tetrahedron (BO_4_) coordination when B atom and O carry on atomic coordination. These complex B-O coordination anions are connected with the Si atom in many ways and become the basic structural unit of borosilicate. This structural information is a key to reveal the stability and activity of B in slag. This key may be lifted by coupling nuclear magnetic resonance spectroscopy (NMR) and Raman spectroscopy studies, where NMR can give information about the short-range order, coordination number, and speciation, and Raman spectroscopy brings knowledge about the ring organization and polymerization of the molten slag [29]. Finally, both approaches provide information about silicate network structure and can be directly correlated to the activity of B in slag.

Authors’ previous work [30] has made some progress about the relationship between the slag structure and the B removal of slag treatment based on the controlling of Na_2_O on optical basicity. However, Al_2_O_3_ is an important component of refining slag, which has been used by many researchers to control the composition of refining slag. Most works have only taken alumina as a basic slag system, and study the effect of other components changes on the removal of B [15,31], lacking attention to the impact of Al_2_O_3_. In a small amount of works, Al_2_O_3_ was used as a component for the oxygen potential controller in the process of slag refining. For example, Barati et al. [17,20] have made some studies about the effect of oxygen potential of the slag on the removal of impurities from Si by varying the SiO_2_/Al_2_O_3_ ratio. Unlike the acidic oxides, only act oxygen acceptors form chemical bonds with other oxides [32]. Al can combine with surrounding oxygen in two forms of coordination in the silicate network. One is a four-coordination form, in which Al^3+^ can replace part of Si and enter the complex anion as a network former [33,34,35]. Besides, Al^3+^ can also exist outside the Si-O backbone in the form of six-coordination, playing the role of cationic network breaker [36]. Therefore, alumina will have more complex effect on the structure of silicate network, as well as the behavior and state of B in network structure. However, much less structural information involving Al_2_O_3_ associated with the refining behavior is available on the coordination environment of network.

In the present work, a SiO_2_–CaO–Al_2_O_3_ system was proposed to remove B in Si. NMR and Raman were used to investigate in detail the degree of B-Si cross linking in a quaternary system SiO_2_–CaO–Al_2_O_3_–B_2_O_3_ slag. Altering the Al_2_O_3_ content and basicity were to control the base structures of SiO_2_–CaO–Al_2_O_3_ glass. Moreover, addition of B_2_O_3_ was carried out to find out the effective base structures that contribute to the formation of B-Si cross linking. In addition, a ternary system SiO_2_–CaO–Al_2_O_3_ slag was proposed to remove B in Si by slag treatment at 1823 K (1550 °C). By comparing the B removal data with the structural behavior of B, we have highlighted the behavior and state of B in the B removal from Si using slag treatment.

## 2. Experimental

### 2.1. Slag Refining

Slag samples were prepared from reagent-grade CaO (99.0 wt% purity metal basis), SiO_2_ (99.5 wt% purity metal basis), and Al_2_O_3_ (99.45 wt% purity metal basis). The CaO-SiO_2_ system was used as base slags, and two groups of slag were set with binary basicity (CaO/SiO_2_ = C/S) 0.6 and 1, respectively. In each group of slag, different Al_2_O_3_ contents (0%, 5%, 10%, 15%, and 20%) were added, and the total mass of every slag was 100 g. Slags were charged into a graphite crucible after blending, and were then heated in a muffle furnace to melt the powdered charge at 1823 K (1550 °C). After heat preservation for 2 h at 1823 K, liquid molten slags were poured onto a cold water to quench. The quenched solid slags were broken, and 10 g slag was crushed to a powder in agate mortar. The powder was sieved to 74 μm using a 200 mesh sieve and sent for X-ray fluorescence (XRF; Axios, PANalytical, Almelo, The Netherlands) verification of composition. The XRF results for the slags are displayed in Table 1.

The matrix Si was prepared from SoG–Si (99.9999 pct mass percent). In order to accurately detect the B content in the refined Si, B in the doped Si was prepared at a concentration of 1000 ppm. The preparation process of the doped Si was as follows: an electromagnetic induction furnace was used to melt 100 g of SoG–Si and 0.1 g high purity B (99.9999 pct mass percent) in a graphite crucible at 1823 K (1550 °C) under an argon atmosphere. Electromagnetic stirring helped to evenly distribute B in the Si. After holding for 1 h at 1823 K (1550 °C), the furnace was cooled to room temperature at a rate of 10 K/s. The Si-doped B was then ground into a powder and inductively coupled plasma mass spectrometry (ICP–MS; iCAP Qc, Thermo Fisher Scientific, Waltham, MA, USA) used to quantify its composition. The 0.5 g slag sample was first accurately weighed on an electronic balance, and then acid-dissolved in a 1:1 mixture of hydrofluoric and nitric acid. The remaining solution was made up to 100 mL, and the content of B was analyzed by ICP. The B content in the Si before slag refining was determined to be 985 ppm (the average value of three replicate samples).

Five grams of doped Si and 30 g pre-melted slag were charged into a molybdenum crucible for each experiment, and the crucible heated to 1823 K (1550 °C) and held for 4 h in an electric resistance furnace under an argon atmosphere. At the end of experiment, the crucible containing the molten Si and slag was quenched in the same stainless steel barrel with liquid nitrogen. Solid Si was ground to a powder for quantifying the B content using ICP–MS. The compositions of metal and slag after the slagging experiments are shown in Table 2. The extents of B removal from Si were obtained by comparing the B contents of the Si before and after refining. The B distribution ratio (*L*_B_) between slag and Si was not examined in this study, because trace B in slag could hardly be measured.

### 2.2. Preparation of Glass Slags

The concentrations of B in the slags after refining were only of the order of hundreds of ppm, which made the analysis of the B structures very challenging. The results of Kline et al. [24] and Sun et al. [26] showed that increasing B_2_O_3_ from 0 to 5% in the slag only increased the relative peak intensities of Raman spectroscopy and ^11^B magic angle spinning (MAS)–NMR spectroscopy, but had no effects on the types of structures. Therefore, a concentration of 3% B_2_O_3_–which would not alter the B structure–was added to the CaO–SiO_2_–Al_2_O_3_ slags to study the structures of B in the refined slags. Six based slags of two groups with different Al_2_O_3_ content (0%, 10%, and 20%) were used to add B_2_O_3_. The preparation process of slags with B_2_O_3_ was the same as described above.

**Figure 1:**
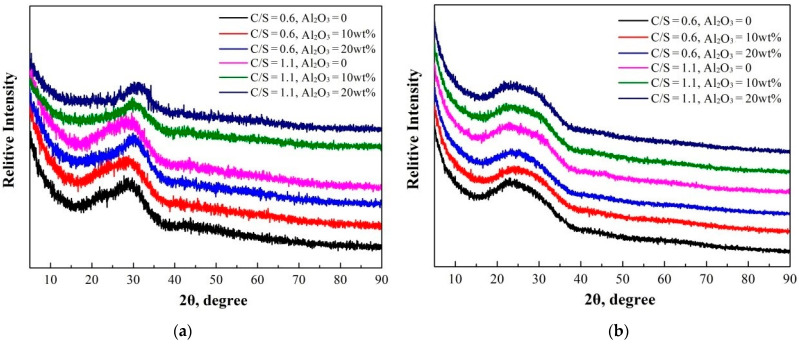
X-ray diffraction results of CaO-SiO_2_-Al_2_O_3_ slags: (**a**) 0 B_2_O_3_ and (**b**) 3 wt% B_2_O_3._

Slags with addition of 3% B_2_O_3_ were also analyzed using X-ray fluorescence, the results as shown in Table 3. X-ray diffractometry (XRD; SmartLab, Rigaku, Japan) was performed to confirm the glassy nature of all samples. As can be seen from Figure 1, these samples were found to have no characteristic XRD peaks, so these slags were judged to be amorphous glass slags [8].

### 2.3. Raman Spectroscopy and ^11^B MAS-NMR Spectroscopy Analysis

To clarify the structural characteristics of borosilicate melts, the two groups of slag were analyzed using a series of spectroscopic techniques. Raman spectrometer analysis was performed on a Horiba Jobin–Yvon HR HR800UV system (Palaiseau, France). A He/Cd laser with an excitation wavelength of 532 nm and a light source of a 1 MW semiconductor was used during the testing process. The spectra of the samples were recorded in the frequency range from 400 to 1800 cm^−1^ at room temperature. The procedure for deconvoluting Raman spectra is a non-linear fitting. Deconvolution of Raman spectra is limited based on the assumptions placed on the procedure.

To further identify the specific structural units in the glasses, solid state ^11^B MAS–NMR measure was performed using a 500 M solid NMR spectrometer (NMR, Karlsruhe, Germany Bruker Avance III HD 500 MHz) with a MAS probe of a 4 mm ZrO_2_ rotor and two pairs of Dupont Vespel caps.

## 3. Results and Discussion

### 3.1. Dependence of L_B_ on Al_2_O_3_ Content and Basicity

**Figure 2:**
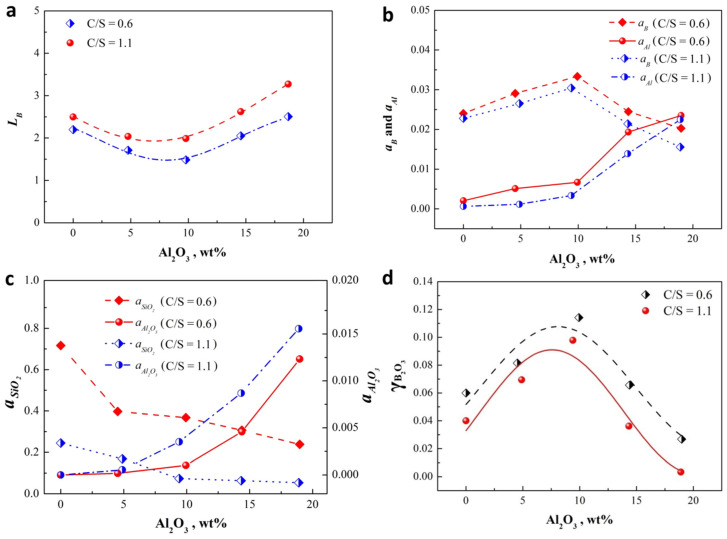
(**a**) B partition ratio between CaO-SiO_2_-Al_2_O_3_ slag and Si phase for different Al_2_O_3_ content and basicity at 1823 K. (**b**) Activity of B and Al in the Si-Ca-Al-B quaternary system at 1823 K. (**c**) Activity of SiO_2_ and Al_2_O_3_ in the CaO-SiO_2_-Al_2_O_3_ ternary system at 1823 K. (**d**) Activity coefficient of B_2_O_3_ for the CaO-SiO_2_-Al_2_O_3_ slag at 1823 K as a function of Al_2_O_3_ content and basicity.

According to the experiment results for the removal of B from Si using CaO-SiO_2_-Al_2_O_3_ slag in Table 2, the B partition ratio (*L*_B_) was obtained by the ratio of B content in slag to that in Si, as shown in Figure 2a. A higher *L*_B_ values are obtained using slag with a basicity of 1.1 than of 0.6 at different Al_2_O_3_ content, which is consistent with most work results [5,13]. The values of *L*_B_ for slags with basicity of 0.6 and 1.1 go down to about 1.5 and 2.0, respectively, comparing to the initial values of 2.2 and 2.5, as Al_2_O_3_ content is near 15 wt%. When Al_2_O_3_ was further added into near 20 wt%, both values increase to more than 2.5 and 3.2, respectively.

Estimation of the B and Al activity in molten Si: The B and Al activity in Si was calculated using the Gibbs-Duhem equation the Si-Ca-Al-B quaternary system. The activity coefficient of B and Al were calculated by Equations (4) and (5).

(4) $$\ln\gamma_{B}=\ln\gamma_{B}^{0}+\varepsilon_{B}^{B}X_{B}+\varepsilon_{B}^{\mathrm{Ca}}X_{\mathrm{Ca}}+\varepsilon_{B}^{\mathrm{Al}}X_{\mathrm{Al}}$$

(5) $$\ln\gamma_{\mathrm{Al}}=\ln\gamma_{\mathrm{Al}}^{0}+\varepsilon_{\mathrm{Al}}^{\mathrm{Al}}X_{\mathrm{Al}}+\varepsilon_{\mathrm{Al}}^{\mathrm{Ca}}X_{\mathrm{Ca}}+\varepsilon_{\mathrm{Al}}^{B}X_{B}$$

Considering the very low concentration of B in Si after refining, the term $\varepsilon_{B}^{B}X_{B}$ and $\varepsilon_{\mathrm{Al}}^{B}$ was ignored [37]. The interaction parameters are available in references [37,38,39], the specific equations are as follows: Equations (6) and (7). The activity of B and Al can be calculated by the product of the activity coefficient and the mole fraction, and these activities in the Si-Ca-Al-B quaternary system at 1823 K are shown in Figure 2b.

(6) $$\begin{array}{c} \ln\gamma_{B\left(l\right)}^{o}=289\left(\pm 450\right)/T+1.19\left(\pm 0.25\right),\;\varepsilon_{B}^{\mathrm{Ca}}=-3.08\pm 0.84,\\ \varepsilon_{B}^{\mathrm{Al}}=-0.7467-9765.9298/T \end{array}$$

(7) $$\begin{array}{c} \ln\gamma_{\mathrm{Al}\left(l\right)}^{o}=-3610/T+0.452,\;\varepsilon_{\mathrm{Al}}^{\mathrm{Ca}}=-0.047,\\ \varepsilon_{\mathrm{Al}}^{\mathrm{Al}}=-40.1+10\times 10^{5}/T \end{array}$$

Calculation of the activity of SiO_2_ and Al_2_O_3_ in molten slag: The SiO_2_ and Al_2_O_3_ activity in the CaO-SiO_2_-Al_2_O_3_ ternary system at 1823 K can be calculated by using FToxid and FTmisc database of Factsage 6.3. These data were arranged for the composition of refining slag used in this study, and the calculated results are shown in Figure 2c. As can be noted, the increase of basicity can lead to the repaid decrease of SiO_2_ activity due to the neutralization of acid oxides and alkaline oxides. For Al_2_O_3_ activity, it presents an increasing trend as basicity increases, which may mean that alumina, as a neutral oxide, exhibits complex changes in different acid and alkaline environments. This complex change is directly related to the behaviors and structures of Al in slag, and will be discussed as follows. As expected, Al_2_O_3_ addition of Al_2_O_3_ promotes the increase of Al_2_O_3_ activity and the relative decrease of SiO_2_ activity.

SiO_2_ and Al_2_O_3_ can provide oxygen potential to oxidize B at the interface [20,32]. Thus, the activity coefficient of B_2_O_3_ can be calculated using the oxidation reaction of B by silica and alumina:

(8) $$\;2B\left(l\right)+\frac{3}{4}{\mathrm{SiO}}_{2}\left(l\right)+\frac{1}{2}{\mathrm{Al}}_{2}O_{3}\left(l\right)=B_{2}O_{3}\left(l\right)+\frac{3}{4}\mathrm{Si}\left(l\right)+\mathrm{Al}\left(l\right)$$

The activity coefficient of B_2_O_3_ can be given by Equation (9):

(9) $$\gamma_{B_{2}O_{3}}=\frac{Ka_{B}^{2}a_{{\mathrm{SiO}}_{2}}^{3/4}a_{{\mathrm{Al}}_{2}O_{3}}^{1/2}}{X_{B_{2}O_{3}}a_{\mathrm{Si}}^{3/4}a_{\mathrm{Al}}}$$

where *K* is the equilibrium constant for Equation (8). The activity of B and Al were obtained from Figure 2b. Figure 2c provides the activity data of SiO_2_ and Al_2_O_3_. The borate molar fraction was obtained from the experimental results in Table 2. The calculated results of the activity coefficient of B_2_O_3_ are plotted in Figure 2d. According to Figure 2a,d, an increase in the amount Al_2_O_3_ results in an opposite change trend of *L*_B_ values comparing with the activity coefficient of B_2_O_3_, which is in agreement with the work of Morita et al. [37]. Thus, investigating the activity coefficient of B_2_O_3_ can reveal the effect of slag on the B removal from Si.

Figure 2d shows that higher basicity (C/S = 1.1) results in an decrease of the B_2_O_3_ activity compared to the basicity 0.6, since acidic oxide B_2_O_3_ interacts with basic oxide CaO. However, Al_2_O_3_ has a complicated influence on the B_2_O_3_ activity. As Al_2_O_3_ content is lower than 10 ± 1 wt%, its increase can lead to the increase of the B_2_O_3_ activity coefficient. This phenomenon is consistent with conventional knowledge that Al_2_O_3_ mainly as an acidic oxide can interact with CaO to release more B_2_O_3_. Beyond the cognitive, an increase in the amount of Al_2_O_3_ more than 10 ± 1 wt% results in a decrease in the B_2_O_3_ activity coefficient. Therefore, it can be inferred that it is not enough to treat B_2_O_3_ and Al_2_O_3_ as a single oxidizing substance, and the state and structure of both in slag must have an important effect on the activity of B.

### 3.2. Role of Al_2_O_3_ in Modifying of the Silicate Network and B Structural

**Figure 3:**
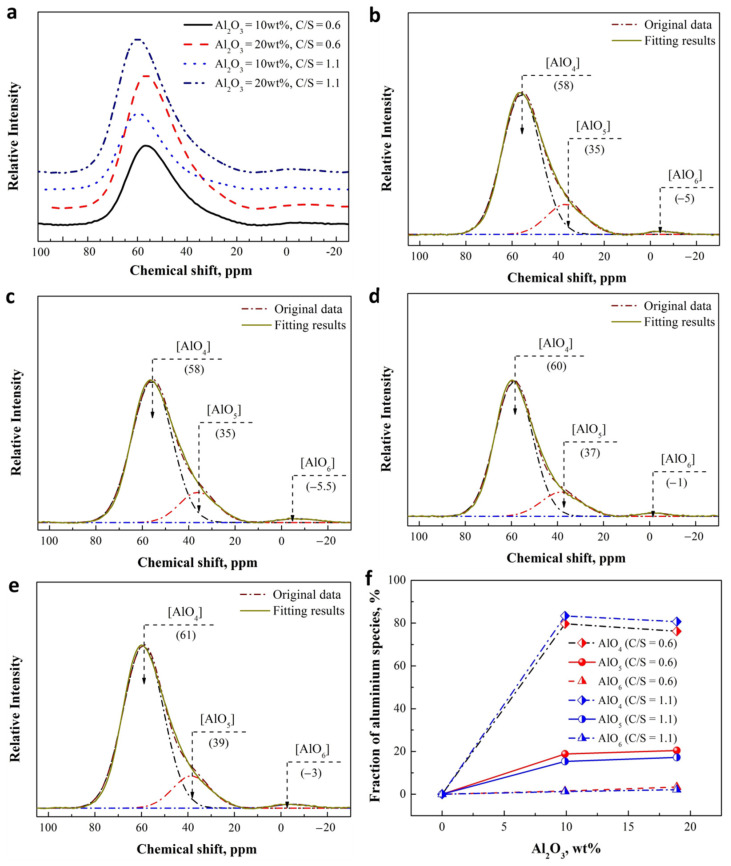
(**a**) ^27^Al NMR MAS spectra of quenched CaO-SiO_2_-Al_2_O_3_ slag at 1823 K as a function of Al_2_O_3_ content and basicity, experimental ^27^Al MAS NMR spectra (solid line), and fitting results (dashed lines with different colors) for CaO-SiO_2_-Al_2_O_3_ slags with 9.93 wt% Al_2_O_3_ and 0.6 of basicity (**b**); 18.97 wt% Al_2_O_3_ and 0.6 of basicity (**c**); 9.4 wt% Al_2_O_3_ and 1.1 of basicity (**d**); 18.9 wt% Al_2_O_3_ and 1.1 of basicity (**e**). (**f**) Fraction of Al species as a function of basicity and Al_2_O_3_ contents based on ^27^Al MAS NMR of the CaO-SiO_2_-Al_2_O_3_ slag.

The existence of alumina has a complex influence on the activity of B, so the role of alumina in the structure of silicate network is required to be understood first. ^27^Al NMR and Raman spectra were preformed to investigate the microstructure of slag. The chemical environments of the Al atoms were examined using ^27^Al MAS NMR on a home-built 500–MHz spectrometer, as shown in Figure 3a. The NMR bands consist of the main contribution, which maximum shifts at approximately 55 to 75 ppm with different Al_2_O_3_ content and basicity, positioning characteristics of the four-fold coordinated Al [AlO_4_] [40,41,42]. An obvious asymmetry–appearing on both sides of the [AlO_4_] characteristic peak for almost all compositions and the bands in lower frequency between 30 ppm and 40 ppm–increases in intensity, and could possibly be related to the characteristic of the five-fold coordinated Al [AlO_5_] [42,43]. A small shoulder around 0 is also visible for the slags with about 20 wt% Al_2_O_3_ and is ascribed to the presence of Al in six-fold coordination, [AlO_6_] [43,44].

The ^27^Al spectra was deconvolved by two symmetric Gaussian functions with the minimum correlation coefficient as *r*^2^ ≥ 0.995 [45], and the fitting results are shown in Figure 3b–e. The resonance of [AlO_4_] four-fold and [AlO_6_] six-fold center is on about 60 ppm and 0. As expected, the bands appearing at near 40 ppm do confirm the existence of the [AlO_5_] five-fold. The relative proportions of these peak areas were calculated to denote the relative abundance of [AlO_4_] four-fold, [AlO_5_] five-fold, and [AlO_6_] six-fold, the results of which are shown in Figure 3f.

^27^Al NMR data indicates that the majority (at least 80%) of Al^3+^ are [AlO_4_] four-fold for the slags with Al_2_O_3_, since the cation compensates Al^3+^ first to form four-fold coordination structure and higher coordination structures can be formed only when the number of cation is insufficient [46]. It can be seen from Figure 3e that the [AlO_4_] population exhibits a decrease as the Al_2_O_3_ increases to about 20 wt%, indicating not enough cation for the charger compensate of Al^3+^. At the same time, the relative amount of [AlO_5_] and [AlO_6_] increases. Although less precisely defined because of a much lower intensity, the population of the [AlO_6_] species also increases to more than 2%. Basicity also brings similar changes to structural units of Al^3+^; Figure 3e shows the obvious increase of [AlO_4_] population as the basicity increasing from 0.6 to 1.1 because the increase in cation can be used for the charger compensation of Al^3+^, which also leads to a reduction in the relative amount of [AlO_5_] and [AlO_6_]. The structure of silicate network has important influence on Al structure, and thus on the ring organization and polymerization of aluminosilicate with varying amounts of Al_2_O_3_ and SiO_2_ using Raman spectroscopy.

**Figure 4:**
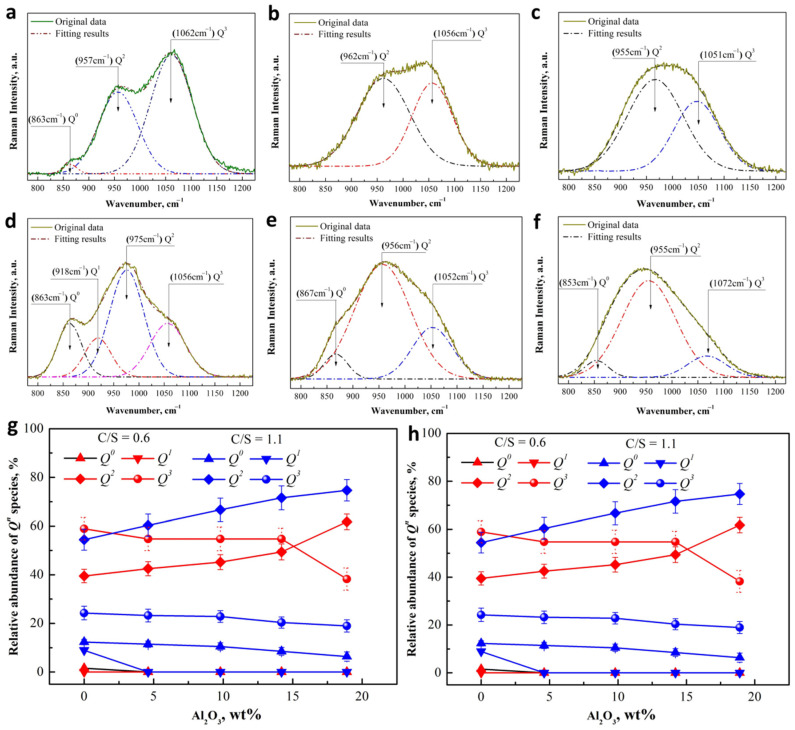
Deconvolution of the Raman spectra for CaO-SiO_2_-Al_2_O_3_ slag at room temperature with 0 Al_2_O_3_ and 0.6 of basicity (**a**); 9.93 wt% Al_2_O_3_ and 0.6 of basicity (**b**); 18.97 wt% Al_2_O_3_ and 0.6 of basicity (**c**); 0 Al_2_O_3_ and 1.1 of basicity (**d**); 9.4 wt% Al_2_O_3_ and 1.1 of basicity (**e**); 18.9 wt% Al_2_O_3_ and 1.1 of basicity (**f**), Distribution of *Q^n^* speciation (**g**), and NBO/T (**h**) as a function of basicity and Al_2_O_3_ contents based on Raman spectroscopy of the CaO-SiO_2_-Al_2_O_3_ slag.

The Raman spectrum of ternary system CaO-SiO_2_-Al_2_O_3_ slags in the high frequency region (800–1250 cm^−1^) were deconvolved using Gauss-deconvolution method with the minimum correlation coefficient as *r*^2^ ≥ 0.995 [45]; the best-fit simulations are shown in Figure 4a–d. For slags with the basicity of 0.6, two bands are obviously observed in the higher frequency region (more than 950 cm^−1^) in Figure 4a–c. Increasing basicity to 1.1 causes more bands to form in the lower frequency region (less than 950 cm^−1^), shown in Figure 4d–f, indicating the increase of the degree of depolymerization of silicate network, in agreement with previous observations [25,26]. A number of works report that the major peaks around 870 cm^−1^, 960 cm^−1^, 990 cm^−1^, and 1050 cm^−1^ could be assigned to *Q*^0^, *Q*^1^, *Q*^2^, and *Q*^3^ [25,26], respectively. The area ratio of an individual species and integral *Q^n^* species were calculated to denote the relative abundance of *Q^n^* (*n* = 1–3) species; the values are plotted in Figure 4g.

Figure 4g shows the change of *Q^n^* speciation as a function of basicity and Al_2_O_3_ content. The degree of polymerization can be represented by the ratio of non-bridging oxygen per tetrahedrally coordinated cation (NBO/T), which can be calculated using Equation (10) [47]. The results as shown in Figure 4h.

NBO/T = 4 × *Q*^0^ + 3 × *Q*^1^ + 2 × *Q*^2^ + 1 × *Q*^3^ (10)

With an increase of basicity from 0.6 to 1.1, more calcium ions are introduced to destroy the network structure, and thus the sheet structure unit (*Q*^3^) population exhibits an obvious decrease accompanied by the increase of structure units with a low degree of polymerization (monomer structure (*Q*^0^), dimer structure (*Q*^1^), and chain structure unit (*Q*^2^)—see Figure 4g—in agreement with previous observations [25,26]. It is noteworthy that for all slags along the Al_2_O_3_ join, the abundance of *Q*^2^ exhibits linear increase, as shown in Figure 4g. The reason for this occurrence may have to do with the characteristics of Al and Si. It has been referred to that Al-O-Si bonds are easier to form than Al-O-Si bonds for Al^3+^ in aluminosilicate than Al-O-Al bonds [40,41,42,43,44]. In the other word, two [AlO_4_] species are difficult to connect due to the unbalanced charge, so in this way, one [SiO_4_] specie must be required to separate them in the middle for the charge to balance. Therefore, it is difficult for [AlO_4_] to exist in silicate structures with an isolated tetrahedron backbone (*Q*^0^ and *Q*^1^), and the substitution of Si for Al is possible in the chain structure (*Q*^2^), sheet structure (*Q*^3^), and framework structure (*Q*^4^).

**Figure 5:**
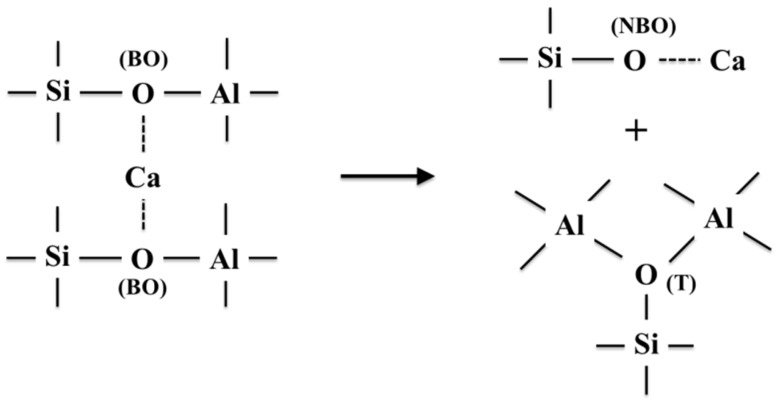
Cartoon of transformation of bridging oxygen (BO) to non-bridging oxygen (NBO) of aluminosilicate.

For slag with basicity of 0.6, less cation limits the degree of the charge balance for Al^3+^, and there will be many structures with a higher coordination ([AlO_5_] and [AlO_6_]) to form. The NBO/T population also presents an increase, as is shown in Figure 3f. Stebbins and Xu [36] have established the transformation mechanism for bridging oxygen (BO) to non-bridging oxygen (NBO) of aluminosilicate, as shown in Figure 5. Based on the characteristic that Al is more likely to replace Si in higher polymer structures, the network structure on the left of the black arrow in Figure 5 tends to represent *Q*^3^ rather than *Q*^2^. According to Stebbins and Xu [36], if incompletely drawn bonds are all bonded to O, all Al remains as AlO_4_, and the new structural unit formed is considered to be a ‘tricluster’. However, if AlO_5_ and AlO_6_ sites are formed, the ‘tricluster’ oxygen (T) may itself behave as an NBO, which would be promoted as Al-O-Al replacing Al-O-Si. Therefore, it can be speculated that the increase of Al_2_O_3_ can promote the formation of ‘tricluster’ oxygen (T), which is used as an NBO to depolymerize *Q*^3^ (Si and Al) and to form *Q*^2^ with more Si-NBO-Ca bonds, see the left of the black arrow in Figure 5. As a result, the relative abundance of *Q*^3^ shows linear decrease as a function of Al_2_O_3_, and more *Q*^2^ forms in this process, as shown in Figure 4g.

In a high-alkaline environment (C/S = 1.1), as shown in Figure 4h, more of the lower polymers *Q*^0^ and *Q*^1^ appear quite clearly, and both structure species follow an overall decrease with increasing Al_2_O_3_ content, especially *Q*^1^. *Q*^2^ population presents a rapid increase as a function of Al_2_O_3_ content, but a slight decrease for *Q*^3^. The reason for this may be that introducing Al_2_O_3_ consumes a certain amount of Ca^2+^ to achieve the charge balance. *Q*^0^ and *Q*^1^ are more likely to lose Ca^2+^ to transform into *Q*^2^ and *Q*^3^ due to the higher concentration of cation, where *Q*^2^ is more likely to be the transformed structure because of the smaller cationic concentration gradient. The increased *Q*^2^ population also proves this speculation. With the slight decrease of *Q*^3^ population, more Al^3+^ exists in tetrahedron form [AlO_4_] due to the sufficient charge compensation of cation, and substitutes some of the Si into the sheet structure (*Q*^3^). Comparatively, fewer higher coordination structures ([AlO_5_] and [AlO_6_]) can destroy *Q*^3^.

In addition, the degree of depolymerization of the silicate network with different Al_2_O_3_ content was further investigated by NBO/T, as shown in Figure 4h. For the slag without Al_2_O_3_, the basicity of 1.1 results in a rapid increase of NBO/T from less than 1.5 to more than 2.2, compared with the lower basicity of 0.6. When Al_2_O_3_ is added, NBO/T of lower basicity slag exhibits a linear increase to more than 2.5, with increasing Al_2_O_3_ content reaching approximately 20 wt%, and higher basicity slag showing a linear decrease as a function of Al_2_O_3_ content. Therefore, it can be believed that the introduction of Al_2_O_3_ can lead to the depolymerization of lower basicity slag (0.6) and the polymerization for higher basicity slag (1.1). This is the same thing for both slags, however, *Q*^2^ population follows an overall increase with increasing Al_2_O_3_ content. These structural changes of aluminosilicate will greatly influence the behavior and state of B in slag, which will be further studied in next section.

**Figure 6:**
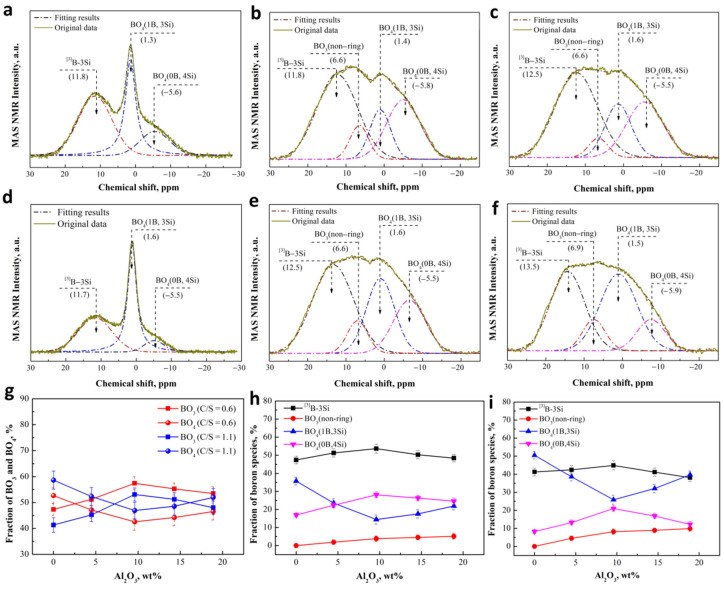
Solid state ^11^B spectra for CaO-SiO_2_-Al_2_O_3_ slags at room temperature with 0 Al_2_O_3_ and 0.6 of basicity (**a**); 9.62 wt% Al_2_O_3_ and 0.6 of basicity (**b**); 19.25 wt% Al_2_O_3_ and 0.6 of basicity (**c**); 0 Al_2_O_3_ and 1.1 of basicity (**d**), 9.16 wt% Al_2_O_3_ and 1.1 of basicity (**e**); 18.73 wt% Al_2_O_3_ and 1.1 of basicity (**f**), (**g**) Fraction of BO_3_ and BO_4_ as a function of basicity and Al_2_O_3_ contents based on Raman spectroscopy of the CaO-SiO_2_-Al_2_O_3_ slag, Fraction of B species as a function of Al_2_O_3_ contents based on Raman spectroscopy of the CaO-SiO_2_-Al_2_O_3_ slag at 0.6 of basicity (**h**) and 1.1 of basicity (**i**).

Unlike silicate network, which has only Si-O tetrahedron, B has an electron configuration of 1s^2^2s^2^2p^1^ outside its nucleus. When B atom and oxygen carry on atomic coordination, the electron layer of B atom can be hybridized to be sp^2^ or sp^3^ orbitals, which present a triangle (BO_3_) or tetrahedron (BO_4_) coordination. In addition, B—oxygen coordination anions are connected with each other in many ways and become the basic structural unit of borate [37,42]. ^11^B MAS-NMR spectra can give the information about the short-range order, coordination number, and speciation, which can be used to identify the structural unit of borate. Figure 6 shows the results of the^11^B MAS-NMR spectra that were deconvolved by two symmetric Gaussian functions with the minimum correlation coefficient as *r*^2^ ≥ 0.995 [39]. The NMR spectra can be divided into two regions overall based on the dividing line of about a 4 ppm chemical shift, where BO_4_ is distributed in lower displacement area, and BO_3_ is located in higher displacement area. Moreover, more information of the specific structural units associated with B atom could be obtained by NMR spectra. BO_3_ trigonal in the networks could be divided into [3] B-3Si and BO_3_ (non-ring), and they are located at approximately 12 ppm and 6.6 ppm in the NMR spectra, respectively [24,27]. BO_4_ tetrahedron is also divided into two structural units, the BO_4_ tetrahedral linked with 1B atom and 3Si atoms (BO_4_ (1B, 3Si)), and the BO_4_ tetrahedral connected to 4Si atoms (BO_4_ (0B, 4Si)), which are located at approximately 1.5 ppm and −5.5 ppm in the NMR spectra [26,27], respectively. An obvious difference for the slags with and without Al_2_O_3_ is that the peaks associated with BO_4_ (1B, 3Si) become less sharp and the characteristic of BO_3_ (non-ring) appears after the addition of Al_2_O_3_. The relative proportions of these peak areas were calculated to denote the relative abundance of the structural units associated with B, and the fraction of total BO_3_ and total BO_4_ as a function of Al_2_O_3_ content and basicity are presented in Figure 6g.

It can be seen from Figure 6g that when the basicity increases from 0.6 to 1.1, the value of BO_4_ population increases by about 5% at the expense of BO_3_ proportion for overall compositions, which is in a good agreement with previous studies [41,42]. Structural transformations that occur in slag upon introduction of alkali oxides into B oxide can be simply explained in that the structure of vitreous B oxide is predominantly formed by layers of boroxol rings composed of BO_3_ triangles. The introduction of alkali oxide into vitreous B oxide leads to the transformation of B-O triangles into B-O tetrahedral BO_4_, the negative charges of which are compensated for by alkali ions [41,42]. For the structural transformations of B structural units due to Al_2_O_3_, Figure 6g shows the trend of BO_4_ population decreases first and then increases as increasing the Al_2_O_3_ content from 0 to about 20 wt% and an opposite trend for the change of BO_3_ proportion. The work of Dhara et al. [41] and DU et al. [42] show that increasing Al_2_O_3_ can results in the decrease of BO_4_, in agreement with the data in Figure 4g when Al_2_O_3_ content increases from 0 to about 10 wt%. It is assumed that to keep the equilibrium of the electric field, the formation of charged ${\mathrm{AlO}}_{4/2}^{-}$ units requires expending four-coordinated [42], resulting in the transformation of BO_4_ to BO_3_. However, as the Al_2_O_3_ content increasing more than 10 wt%, the increase of BO_4_ may be attributed to the modification of silicate network by Al_2_O_3_. As shown in the above results in Figure 6g, the increase of Al_2_O_3_ leads to an increase of *Q*^2^ (Si) by the depolymerization of *Q*^3^ (Si) for the slags with a basicity of 0.6 and 1.1. The NBO of *Q*^2^ (Si) can be used to transform BO_3_ to BO_4_ like the cation, which may play a major role as the Al_2_O_3_ increases to more than 10 wt%.

The specific structural units associated with B atom are further investigated, as shown in Figure 6h,i. As can be noted, for the slag with a basicity of 0.6, when there is no alumina in the slag, the [3] B-3Si population is the largest, accounting for almost half of the total. The fraction of BO_4_ (1B, 3Si) and BO_4_ (0B, 4Si) is about 35% and 16%, respectively. As the Al_2_O_3_ increases to about 10 wt%, the proportion of alumina increases gradually along with the BO_3_ (non-ring) population. On the contrary, BO_4_ (1B, 3Si) proportion decreases rapidly from about 35% to 10%. When Al_2_O_3_ was added to more than about 10 wt%, rapid increase appears as the expense of BO_4_ (0B, 4Si) and [3] B-3Si. In a higher alkaline environment (C/S = 1.1), B species presents the same change trend as the slag with a basicity of 0.6, except for the higher fraction of BO_4_ (1B, 3Si).

**Figure 7:**
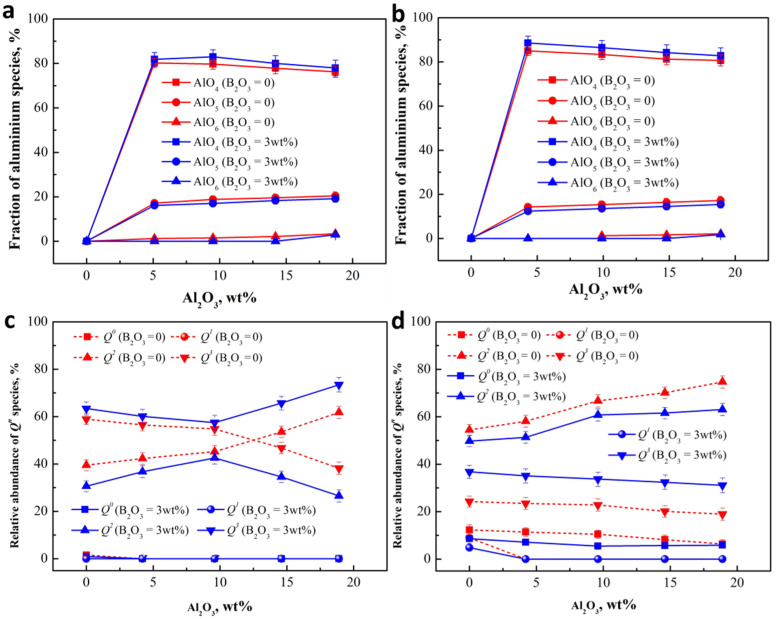
Fraction of Al species as a function of basicity and Al_2_O_3_ contents based on ^27^Al MAS NMR of the CaO-SiO_2_-Al_2_O_3_ slag at 0.6 of basicity (**a**) and 1.1 of basicity (**b**). Distribution of *Q^n^* speciation as a function of Al_2_O_3_ contents based on Raman spectroscopy of the CaO-SiO_2_-Al_2_O_3_ slag at 0.6 of basicity (**c**) and 1.1 of basicity (**d**).

The investigation on the changes of alumina structural units after B oxide addition can help to understand the combination of B and Al, as shown in Figure 7a,b. As can be noted, the addition of B_2_O_3_ can lead to the decrease of AlO_5_ and AlO_6_ for all compositions, so the AlO_4_ population increases relatively. The work of Du et al. [42] showed that Al_2_O_3_ could improve the compatibility of B-O network and Si-O network by forming B-O-Al-O-Si bonds, where B-O-Al bonds are more likely to belong to the BO_3_ that is stronger than BO_4_. Moreover, Stebbins et al. [36] put forward that if AlO_5_ or AlO_6_ sites are formed, the “tricluster” oxygen (T) may itself behave as a NBO. Previous studies have shown that NBO could promote the bonding of B to the network. Therefore, it can be believed that B can combine with NBO of AlO_5_ or AlO_6_ to form B-O-Al-O-Si bonds, which has a great effect on trapping B.

The influence of B_2_O_3_ addition on the change of Raman structures of aluminosilicate was used to study the way of B entering the network, as shown in Figure 7c,d. It can be observed that the decrease of alumina *Q*^2^ population is accompanied by the increase of *Q*^3^ fraction as increasing Al_2_O_3_ content. For slag with a basicity of 0.6 in Figure 7c, the decrease of *Q*^2^ increased obviously when Al_2_O_3_ content was more than about 10 wt%. This trend is the same as that of the higher basicity slag (Figure 7b), although amplitude of increase of the latter is relatively low. Moreover, for the higher basicity slag, B_2_O_3_ addition also leads to the decrease of *Q*^0^ and *Q*^1^ fraction, in agreement with the previous work. It is noteworthy that a smaller change of the fraction of *Q*^0^ and *Q*^1^ appears when slag contains about 20 wt%, indicating that the effect of B_2_O_3_ on *Q*^0^ and *Q*^1^ decreases gradually with the increase of alumina content. As discussed in Section 3.2, Al is easier to insert into the *Q*^3^ structural unit, and the *Q*^2^ is mainly silicate structure. B will combine with NBO in *Q*^2^ to form *Q*^3^ structural unit, but the degree of binding mainly depends on BO_4_ (1B, 3Si) rather than BO_3_. It can be speculated that a significant reduction of *Q*^2^ population may be attribute to the increase in BO_4_ (1B, 3Si) fraction when Al_2_O_3_ content increases to about 20 wt%.

### 3.3. Discussion on the Role of B Structural in Aluminosilicate in B Removal

The difference of the chemical potential of B impurity in Si melt and slag is the driving force of the oxidation of B from silicon into slag, and the activity is an important parameter to quantify the chemical potential. The activity of B depends on the composition of the system, whether Si melt or slag. For silicon purification, the content of other impurities except B in silicon melt is low, so the activity of B in Si melt only changes to a limited degree. Therefore, the activity of B in slag will play a leading role in the oxidation removal of B due to the complexity of slag composition.

**Figure 8:**
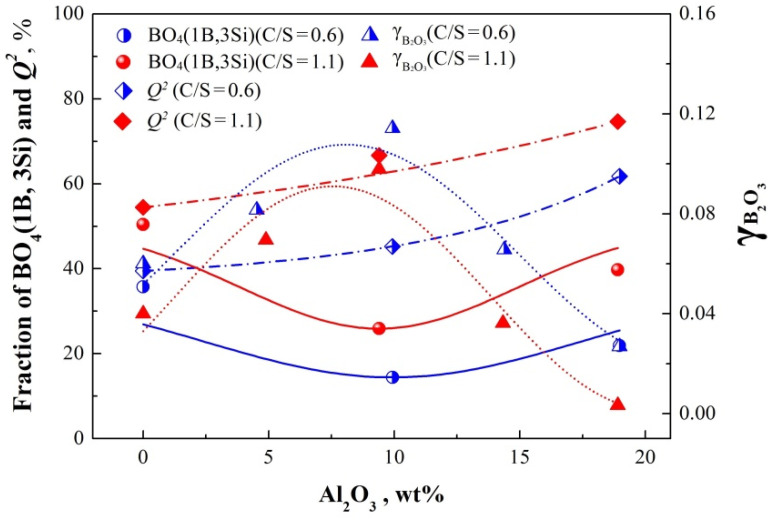
Comparison of the $\gamma_{B_{2}O_{3}}$ value and structural units (*Q*^2^ and BO_4_ (1B, 3Si)) under the condition of different Al_2_O_3_ content and basicity.

According to the above results and discussions, *Q*^2^ structural unit and BO_4_ (1B, 3Si) species have the important influence on B fixation in slag. Therefore, the comparison between the activity coefficient of B and the fraction of *Q*^2^ and BO_4_ (1B, 3Si) was carried out to discuss the role of B structural in aluminosilicate in B removal, as shown in Figure 8. As can be noted, the value of $\gamma_{B_{2}O_{3}}$ first increased then decreased with the increase of the Al_2_O_3_ content from 0 to 20 wt%, and it reached its maximum when the Al_2_O_3_ content was about 10 wt%. When the Al_2_O_3_ content in the slag is below the concentration range of 10 ± 1 wt%, increasing the Al_2_O_3_ content will lead to a reduction in the BO_4_ (1B, 3Si) population, indicating that the binding ability between B and aluminosilicate network is gradually weakened. In other words, the ability of B to live in the slag increases, thus the activity coefficient of B in the slag shows the trend of increasing. At the same time, the fraction of *Q*^2^ also shows an increasing trend, indicating that the oxidation capacity of the slag to B increases at the interface. As a result, more B enters the slag and does not effectively combine with the silicate network, thus promoting the increase of the activity coefficient of B in the slag.

When the Al_2_O_3_ content is greater than 10 ± 1 wt%, however, B is more easily incorporated into the aluminosilicate network due to the formation of more *Q*^2^ and BO_4_ (1B, 3Si). Both structural forms are favorable for incorporating B, which is also proved based on the results that the activity coefficient of B decreased rapidly as increasing Al_2_O_3_ content from 10 ± 1 wt% to about 20 wt%. Finally, the degree of B removal also increased rapidly.

## 4. Conclusions

To solve the problem of impurity deep separation in the preparation of high purity Si, the structure-activity relationship between slag structure and B impurity was studied to clarify the B removal mechanism of slag refining from Si. The leading structure contributing to B impurity removal was found in the SiO_2_–CaO–Al_2_O_3_–B_2_O_3_ slag, which provided theoretical guidance for design of refining slag based on structural modifications associated with the B removal for SoG-Si. Al_2_O_3_ were selected for slag structural modifications because that alumina has both acid and alkaline oxide properties. The introduction of Al_2_O_3_ can lead to the depolymerization of lower basicity slag (0.6) and the polymerization for higher basicity slag (1.1). Accordingly, the values of *L*_B_ first show a reduction, and then an increase in Al_2_O_3_ content. *Q*^2^ structural unit is an important site for capturing B. BO_4_ (1B, 3Si) species are the main form of connection between B and silicate networks, which contributes to the reduction of B activity. *Q*^2^ population of both slags with basicity of 0.6 and 1.1 show an overall increase with increasing Al_2_O_3_ content. The Fraction of BO_4_ (1B, 3Si) decreases first and then increases with the addition of Al_2_O_3_ to about 20 wt%.

The role of slag structure in the B activity can be explained as follows: when Al_2_O_3_ content is less than 10 ± 1 wt%, the decrease of BO_4_ (1B, 3Si) population plays a major role in deteriorating the connectivity between B and aluminosilicate network, which leads to a higher activity of B. When the Al_2_O_3_ content is greater than 10 ± 1 wt%, B is easier to incorporate into the silicate network due to the formation of more *Q*^2^ and BO_4_ (1B, 3Si), which contributes to a rapid decline in activity of B in slag.

## Figures and Tables

**Table 1:** Pre-melting slag components without B_2_O_3_, as determined from X-ray fluorescence and inductively coupled plasma analysis, wt%.

CaO	SiO_2_	Al_2_O_3_	C/S
36.98	58.73	0	0.63
36.05	58.15	4.52	0.62
33.43	52.24	9.93	0.64
31.09	50.15	14.38	0.62
31.12	47.89	18.97	0.65
52.69	48.79	0	1.08
50.52	46.35	4.89	1.09
46.88	43.01	9.40	1.09
44.33	40.30	14.33	1.10
41.49	38.78	18.90	1.07

**Table 2:** Components of slag and Si after 4 h at 1823 K, (mass%, mass ppm).

C/S	Slag				Molten Si		
	CaO	SiO_2_	Al_2_O_3_	(B) (Mass ppm)	[B] (Mass ppm)	[Ca] (Mass ppm)	[Al] (Mass ppm)
0.63	38.68	61.32	0	134.17	61.00	4036.00	48.60
0.69	39.02	56.21	4.77	131.53	76.80	1776.60	232.20
0.67	36.41	53.8	9.79	129.77	87.40	529.20	303.60
0.66	34.24	51.2	14.56	133.49	65.08	710.40	851.40
0.71	33.71	47.61	18.68	135.33	54.00	597.00	983.80
1.02	50.44	49.56	0	160.06	64.10	3638.2	28.40
1.04	48.64	46.78	4.58	167.07	82.00	4874.2	53.40
1.19	49.45	41.39	9.16	144.87	72.96	7284.2	153.20
1.16	46.04	39.71	14.25	154.51	58.94	5886.2	619.20
1.16	43.98	37.85	18.17	140.08	42.80	4648.2	981.40

**Table 3:** Pre-melting slag components with B_2_O_3_, as determined from X-ray fluorescence and inductively coupled plasma analysis, mass%.

CaO	SiO_2_	Al_2_O_3_	B_2_O_3_	C/S
35.42	61.75	0	2.83	0.57
36.84	55.82	4.53	2.81	0.66
34.36	53.23	9.62	2.79	0.64
28.61	52.98	15.61	2.80	0.54
29.74	48.20	19.25	2.81	0.62
50.81	46.41	0	2.78	1.09
47.98	44.43	4.78	2.81	1.08
45.89	42.15	9.16	2.80	1.09
42.32	39.92	14.97	2.79	1.06
40.99	37.51	18.73	2.77	1.09

## Data Availability

All data in this paper are obtained from our experiment, and are authentic and reliable. The publication of data has obtained the consent of all authors.

## References

[1] Selvaraj P., Baig H., Mallick T.K., Siviter J., Montecucco A., Li W., Paul M., Sweet T., Gao M., Knox A.R. (2018). Enhancing the efficiency of transparent dye-sensitized solar cells using concentrated light. Solar Energy Materials and Solar Cells. Sol. Energy Mater. Sol. Cells.

[2] Ganesamoorthy R., Sathiyan G., Sakthivel P. (2017). Fullerene based acceptors for efficient bulk heterojunction organic solar cell applications. Solar Energy Materials and Solar Cells. Sol. Energy Mater. Sol. Cells.

[3] Schmidt J., Aberle A.G., Hezel R. (1997). Investigation of Carrier Lifetime Instabilities in Cz-Grown Silicon. Proceedings of the 26th IEEE Photovoltaic Specialists Conference.

[4] Woditsch P., Koch W. (2002). Solar grade silicon feedstock supply for PV industry. Energy Mater. Sol. Cells.

[5] Peng M., Shi B., Han Y., Li W., Zhang J. (2022). Crystal facet dependence of SiHCl_3_ reduction to Si mechanism on silicon rod. Api. Surf. Sci..

[6] Johnston M.D., Khajavi L.T., Li M., Sokhanvaran S., Barati M. (2012). High-temperature refining of metallurgical-grade silicon: A review. JOM.

[7] Safarian J., Tangstad M. (2012). Vacuum refining of molten silicon. Metall. Mater. Trans. B.

[8] Kim G.H., Sohn I. (2014). Role of B_2_O_3_ on the viscosity and structure in the CaO-Al_2_O_3_-Na_2_O-based system. Metall. Mater. Trans. B.

[9] Zhu Y., Wu J., Wang Q., Ma W., Wei K., Lei Y. (2020). Impurity removal from diamond-wire cutting waste by slag refining and electromagnetic stirring. JOM.

[10] Qian G., Sun Y., Wang Z., Wei K., Ma W. (2021). Novel application of electroslag remelting refining in the removal of boron and phosphorus from silicon alloy for silicon recovery. ACS Sustain. Chem. Eng..

[11] Xu M., Zhu Y., Wu J., Xia Z., Wei K., Ma W. (2021). Mechanism of boron removal using calcium silicate slag containing CaCl_2_ under O_2_ atmosphere. Metall. Mater. Trans. B.

[12] Li P., Wang K., Fang M., Zhang L., Jiang D.C., Li J.Y., Tan Y. (2018). Boron removal from silicon by slag refining using Na_2_O-SiO_2_ in industrial applications. Sep. Sci. Technol..

[13] Chen H., Morita K., Ma X., Chen Z., Wang Y. (2019). Boron removal for solar-grade silicon production by metallurgical route: A review. Sol. Energy Mater. Sol. Cells.

[14] Fang M., Lu C., Huang L., Lai H., Chen J., Li J., Ma W., Xing P., Luo X. (2014). Effect of Calcium-Based Slag Treatment on Hydrometallurgical Purification of Metallurgical-Grade Silicon. Ind. Eng. Chem. Res..

[15] Chen G., Li Y., Huang L., Zhang C., Luo X. (2022). High-value recycling of photovoltaic silicon waste: Accelerated removal of impurity boron through Na_3_AlF_6_-enhanced slag refining. Sep. Sci. Technol..

[16] White J.F., Du S. (2014). Mass transfer in slag refining of silicon with mechanical stirring: Transient interfacial phenomena. Metall. Mater. Trans. B.

[17] Zhou Q., Wen J., Wu J., Ma W., Xu M., Wei K., Zhang Z., Zhang L., Xu J. (2019). Recovery and purification of metallic silicon from waste silicon slag in electromagnetic induction furnace by slag refining method. J. Clean. Prod..

[18] Ma X., Yoshikawa T., Morita K. (2014). Purification of metallurgical grade Si combining Si–Sn solvent refining with slag treatment. Sep. Purif. Technol..

[19] Teixeira L.A.V., Morita K. (2009). Removal of boron from molten silicon using CaO–SiO_2_ based slags. ISIJ Int..

[20] Johnston M.D., Barati M.J. (2011). Effect of slag basicity and oxygen potential on the distribution of boron and phosphorus between slag and silicon. J. Non-Cryst. Solids.

[21] Peitl O., Zanotto E.D., Serbena F.C., Hench L.L. (2012). Compositional and microstructural design of highly bioactive P_2_O_5_–Na_2_O–CaO–SiO_2_ glass-ceramics. Acta Biomater..

[22] Ye H., Xin S., Yin Y., Li J., Guo Y., Wan L. (2017). Stable Li plating/stripping electrochemistry realized by a hybrid Li reservoir in spherical carbon granules with 3D conducting skeletons. J. Am. Chem. Soc..

[23] Liu L., Yin Y., Li J., Wang S., Guo Y., Wan L. (2018). Uniform LithiumNucleation/Growth Induced by Lightweight Nitrogen-Doped Graphitic Carbon Foams for High-Performance Lithium Metal Anodes. Adv. Mater..

[24] Du L., Stebbins J.F. (2003). Solid-state NMR study of metastable immiscibility in alkali borosilicate glasses. J. Non-Cryst. Solids.

[25] Wang L., Wang Y., Wang Q., Chou K. (2016). Raman Structure Investigations of CaO-MgO-Al_2_O_3_-SiO_2_-CrO_x_ and Its Correlation with Sulfide Capacity. Metall. Mater. Trans. B.

[26] Sun Y., Zhang Z. (2015). Selective crystallization behavior of CaO-SiO_2_-Al_2_O_3_-MgO-FetO-P_2_O_5_ steelmaking slags modified through P_2_O_5_ and Al_2_O_3_. Metall. Mater. Trans. B.

[27] Du L., Stebbins J.F. (2003). Nature of silicon-boron mixing in sodium borosilicate glasses: A high-resolution ^11^B and ^17^O NMR study. J. Phys. Chem. B.

[28] Tilocca A., Cormack A.N. (2007). Structural effects of phosphorus inclusion in bioactive silicate glasses. J. Phys. Chem. B.

[29] Novikov A.N., Neuville D.R., Hennet L., Gueguen Y., Thiaudière D., Charpentier T., Florian P. (2017). Al and Sr environment in tectosilicate glasses and melts: Viscosity, Raman and NMR investigation. Chem. Geol..

[30] Qian G., Wang Z., Gong X., Sun L. (2017). The importance of slag structure to boron removal from silicon during the refining process: Insights from Raman and nuclear magnetic resonance spectroscopy study. Metall. Mater. Trans. B.

[31] Maramba B., Eri R.H. (2008). Phosphide capacities of ferromanganese smelting slags. Miner. Eng..

[32] Johnston M.D., Barati M. (2010). Distribution of impurity elements in slag–silicon equilibria for oxidative refining of metallurgical silicon for solar cell applications. Sol. Energy Mater. Sol. Cells.

[33] Wang S., Scrivener K.L. (2003). 29Si and 27Al NMR study of alkali-activated slag. Cement Concr. Res..

[34] Lee S.K., Stebbins J.F. (2000). Al–O–Al and Si–O–Si sites in framework aluminosilicate glasses with Si/Al = 1: Quantification of framework disorder. J. Non-Cryst. Solids.

[35] Losq C.L., Neuville D.R., Florian P., Henderson G.S., Massiot D. (2014). The role of Al^3+^ on rheology and structural changes in sodium silicate and aluminosilicate glasses and melts. Geochim. Cosmochim. Acta.

[36] Stebbins J.F., Xu Z. (1997). NMR evidence for excess non-bridging oxygen in an aluminosilicate glass. Nature.

[37] Teixeira L.A.V., Morita K. (2009). Behavior and State of Boron in CaO–SiO_2_ Slags during Refining of Solar Grade Silicon. ISIJ Int..

[38] Ma X., Yoshikawa T., Morita K. (2013). Removal of boron from silicon-tin solvent by slag treatment. Metall. Mater. Trans. B.

[39] Yoshikawa T., Morita K. (2005). Thermodynamic property of B in molten Si and phase relations in the Si–Al–B system. Mater. Trans..

[40] Peeters M.P.J., Kentgens A.P.M. (1997). A ^27^A1MAS, MQMAS and off-resonance nutation NMR study of aluminium containing silica-based sol-gel materials. Solid State Nucl. Mag..

[41] Dhara A., Mishra R.K., Shukla R., Valsala T.P., Sudarsan V., Tyagi A.K.C., Kaushik P. (2016). A comparative study on the structural aspects of sodium borosilicate glasses and barium borosilicate glasses: Effect of Al_2_O_3_ addition. J. Non-Cryst. Solids.

[42] Du W.F., Kuraoka K., Akai T., Yazawa T. (2000). Study of Al_2_O_3_ effect on structural change and phase separation in Na_2_O-B_2_O_3_-SiO_2_ glass by NMR. J. Mater. Sci..

[43] Neuville D.R., Cormier L., Montouillout V., Massiot D.J. (2007). Local Al site distribution in aluminosilicate glasses by 27Al MQMAS NMR. J. Non-Cryst. Solids.

[44] Goldbourt A., Landau M.V., Vega S. (2003). Characterization of aluminum species in alumina multilayer grafted MCM-41 using 27Al FAM (II)-MQMAS NMR. J. Phys. Chem. B.

[45] Zhang P., Grandinetti P.J., Stebbins J.F. (1997). Anionic species determination in CaSiO_3_ glass using two-dimensional ^29^Si NMR. J. Phys. Chem. B.

[46] Zhang G., Zheng W., Chou K. (2017). Influences of Na_2_O and K_2_O additions on electrical conductivity of CaO-MgO-Al_2_O_3_-SiO_2_ melts. Metall. Mater. Trans. B.

[47] Kline J., Tangstad M., Tranell G. (2015). A Raman spectroscopic study of the structural modifications associated with the addition of calcium oxide and boron oxide to silica. Metall. Mater. Trans. B.

